# Circulating Osteoprotegerin Levels Independently Predict All-cause Mortality in Patients with Chronic Kidney Disease: a Meta-analysis

**DOI:** 10.7150/ijms.34274

**Published:** 2019-09-07

**Authors:** Qing-xiu Huang, Jian-bo Li, Xiao-wen Huang, Lan-ping Jiang, Lin Huang, Hai-wen An, Wen-qin Yang, Jie Pang, Yan-lin Li, Feng-xian Huang

**Affiliations:** 1 Department of Nephrology, Zhongshan Hospital of Traditional Chinese Medicine, Affiliated to Guangzhou University of Chinese Medicine, Zhongshan, People's Republic of China; 2 Department of Nephrology, The First Affiliated Hospital, Sun Yat-sen University, Guangzhou, People's Republic of China; 3 Key Laboratory of Nephrology, National Health Commission and Guangdong Province, People's Republic of China; 4 Department of Ultrasonography, Zhongshan Hospital of Traditional Chinese Medicine, Affiliated to Guangzhou University of Chinese Medicine, Zhongshan, People's Republic of China

**Keywords:** osteoprotegerin, all-cause mortality, chronic kidney disease, meta-analysis

## Abstract

**Background**: Studies have shown inconsistent results regarding the association between circulating osteoprotegerin (OPG) levels and all-cause mortality in patients with chronic kidney disease (CKD). The aim of this meta-analysis is to investigate the association between circulating OPG levels and all-cause mortality in patients with CKD.

**Methods**: The PubMed, EMBASE and Cochrane Library databases were searched for eligible studies investigating the association between circulating OPG levels and all-cause mortality in patients with CKD. Pooled hazard ratios (HRs) and the corresponding 95% confidence intervals (CIs) were calculated using a random effects model.

**Results**: In all, 13 studies that included 2,895 patients with CKD were included in this analysis. According to the meta-analysis, patients with the highest circulating OPG level had a significantly higher risk of all-cause mortality (7 studies; the adjusted HR, 1.88; 95% CI, 1.45 - 2.44) compared with patients with the lower circulating OPG level. An increase of 1 pmol/L in the circulating OPG level was associated with a 6% increased risk of all-cause mortality (7 studies; the adjusted HR, 1.06; 95% CI, 1.03-1.10). A subgroup analysis by dialysis methods suggested that an elevated circulating OPG level was independently associated with all-cause mortality in the HD only population.

**Conclusion**: Elevated circulating OPG levels independently predict an increased risk of all-cause mortality in patients with CKD, especially in the HD only population.

## Introduction

Chronic kidney disease (CKD) is an increasing global public health issue. Currently, the literature has reported an estimated prevalence of CKD of 10.8-13.6% in adults 1-3. Patients with CKD demonstrate a higher risk of mortality than the general population 4. Previous studies have identified many risk factors for mortality in CKD patients, such as smoking, anaemia, left ventricular hypertrophy and high blood pressure 5-7. In addition, some published research has suggested the potential value of other nontraditional risk factors including circulating osteoprotegerin (OPG) levels 8-10.

OPG is a soluble tumour necrosis factor (TNF) superfamily receptor 11. It inhibits the actions of the cytokine receptor activator of nuclear factor kappa-B ligand (RANKL) and TNF-related apoptosis-inducing ligand (TRAIL) by preventing their binding to signalling receptors in the cell membrane 12. Inhibition of the RANK/ TRAIL pathway results in less osteoclast differentiation as well as reduced activation and survival of mature osteoclasts 13. OPG is also involved in metabolic bone disease and plays a potential role in the prognosis of CKD 14. Several studies 8, 9, but not all 15, 16, have suggested a significant association between OPG levels and all-cause mortality in patients with CKD. However, there is conflicting evidence as to whether an elevated circulating OPG level is an independent risk factor for all-cause mortality in participants with CKD.

We hypothesized that an elevated circulating OPG level was an independent predictor of all-cause mortality in patients with CKD. Therefore, we performed a qualitative and quantitative meta-analysis of all available studies that reported the association of OPG levels with all-cause mortality in patients with CKD.

## Methods

### Literature search

This meta-analysis was conducted in accordance with the Preferred Reporting Items for Systematic Reviews and Meta-Analyses (PRISMA) statement and is registered with the International Prospective Register of Systematic Reviews (number CRD42018092797) 17.

We searched for relevant studies published between January 1970 and December 2018 in the PubMed, EMBASE and Cochrane Library databases. We used the search terms "osteoprotegerin" and "kidney". The complete search used for PubMed was ("Osteoprotegerin"[Mesh] OR "Osteoprotegerin" [All Fields] OR "OPG" [All Fields] OR "OCIF Protein" [All Fields] OR "Osteoclastogenesis Inhibitory Factor" [All Fields] OR "Tumor Necrosis Factor Receptor 11b" [All Fields]) AND ("Renal" [All Fields] OR "Kidney" [All Fields] OR "Dialysis" [All Fields] AND ("Mortality" [All Fields] OR "death" [All Fields] OR "survival" [All Fields] OR "prognosis" [All Fields] OR "outcome" [All Fields]). We also performed a manual search using the reference lists of key articles published in English. The search process was performed and confirmed by two investigators (Q.X.H. and J.B.L.).

### Research selection

We regarded studies as eligible if they met all the following criteria: (1) circulating OPG levels were measured at baseline; (2) all-cause mortality was the main outcome; (3) the studies enrolled adult patients with CKD, which was defined according to the KDOQI guideline 18; and (4) the studies had available data on adjusted hazard ratios (HRs) and their corresponding 95% confidence intervals (CIs) (or provided the data needed to calculate them) for all-cause mortality associated with a 1 pmol/L increase in the circulating OPG level or they compared high and low circulating OPG levels. The circulating OPG level groups were based on the definitions used in each study. No restriction was made with regard to language, and published abstracts were also considered. Two reviewers (X.W.H. and L.H.) independently screened the studies and selected the articles. In cases of disagreement, a consensus was reached through discussion with the senior author (F.X.H.). Corresponding authors were emailed to obtain additional data for the eligible articles if the relevant data were not reported.

### Data extraction and quality assessment

Two investigators (H.W.A. and W.Q.Y.) extracted the following data from each included study using standardized forms: author, publication year, research population, dialysis method, patient number, number of males, age of the research population, circulating OPG concentration, follow-up duration and the number of deaths. The most fully adjusted HRs with 95% CIs were extracted from all the eligible studies. One senior author (L.P.J.) supervised the entire data extraction process. The quality of the studies was evaluated by consensus between the two investigators (J.P. and Y.L.L.) in accordance with the Newcastle-Ottawa Scale (NOS) (maximum score, 9) 19. The overall research quality was defined as poor (score 0-3), fair (score 4-6), or high (score 7-9).

### Statistical analysis

The relationship between circulating OPG levels and all-cause mortality was summarized by considering circulating OPG not only as a categorical variable (comparing the highest to the lower circulating OPG levels) but also as a continuous variable (investigating the change in all-cause mortality for every 1 pmol/L increase in the level of circulating OPG). Each HR was transformed to its natural logarithm (log HR), and the variance for each log HR was calculated from its corresponding 95% CI. Random effects models were used to obtain the pooled log HR, and the overall HR and its 95% CI were calculated by exponentiation of the pooled log HR 20.

We used Stata (version 12.0) for all statistical analyses. Statistical tests were two-sided and used a significance level of p < 0.05. We used the Cochran *Q* test to assess heterogeneity among studies 21. We also performed the *I*² test to assess the magnitude of the heterogeneity between studies, with values ≤ 40%, 40 - 75% and ≥ 75% regarded as indicating low, moderate and high degrees of heterogeneity, respectively 21-23. A subgroup analysis was conducted to assess the effects between populations that underwent different dialysis methods. A sensitivity analysis was performed to explore the impact of each individual study by removing one study at a time.

## Results

### Literature search and study characteristics

**Fig 1:**
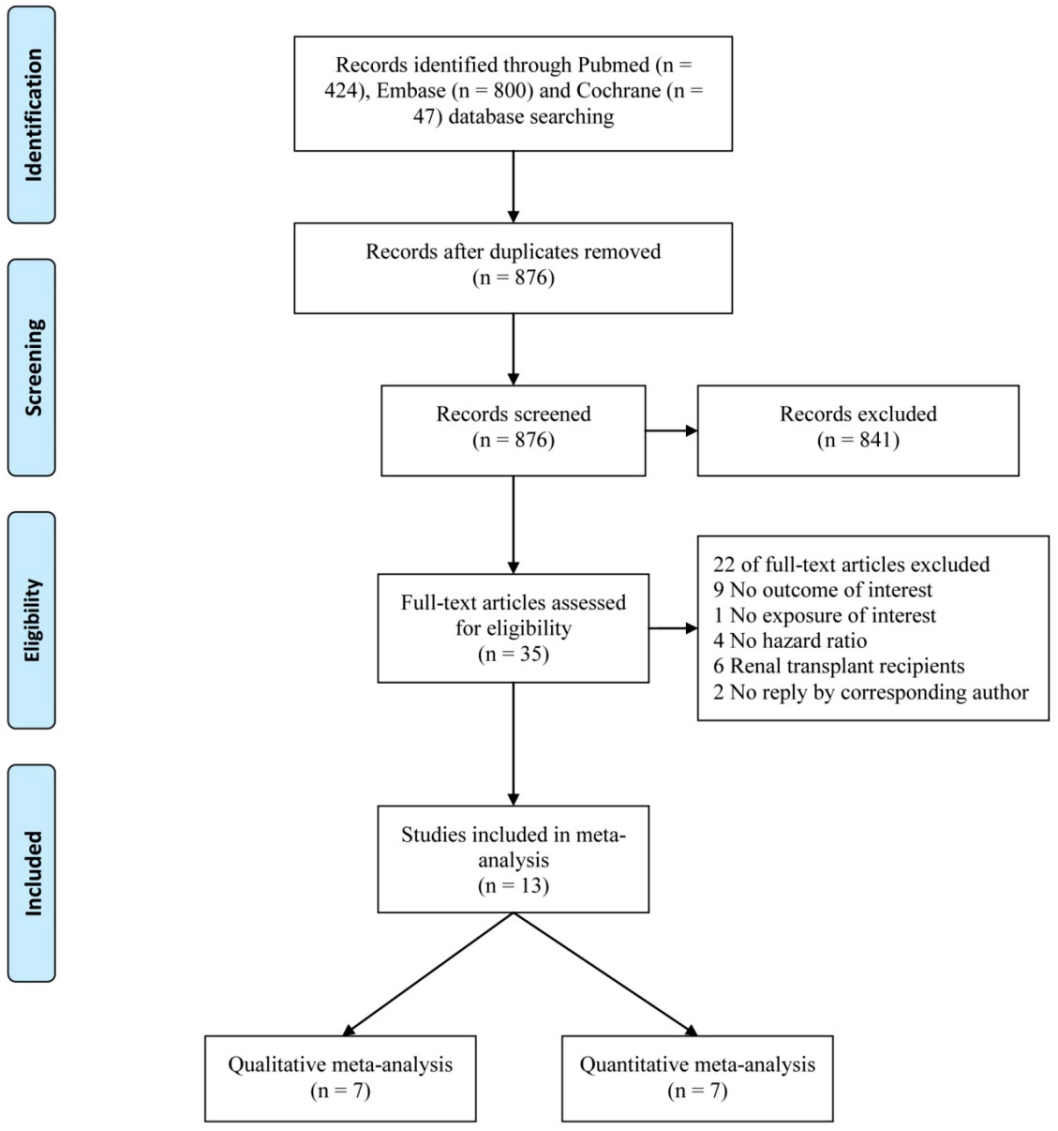
Flow chart of study selection

In all, 876 non-duplicated potential studies were identified, and 13 8-10, 15, 24-32 were finally included in the meta-analysis (**Fig. 1**). Seven studies 24, 26-28, 30-32 were included in a qualitative meta-analysis to assess the association of the circulating OPG level, as a categorical variable, with all-cause mortality. Seven studies 8-10, 15, 25, 27, 29 were included in a quantitative meta-analysis to assess the association of a 1 pmol/L increase in the circulating OPG level with all-cause mortality. The eligible studies were published from 2006 to 2018. The characteristics and quality scores of the included studies are displayed in **Table 1**. In total, 2,895 individuals were included, and 1,257 deaths were recorded. All studies were considered to have fair (scale of 5-6) to high (scale of 7-9) quality.

### Association of the circulating OPG level, as a categorical variable, with all-cause mortality

**Fig 2:**
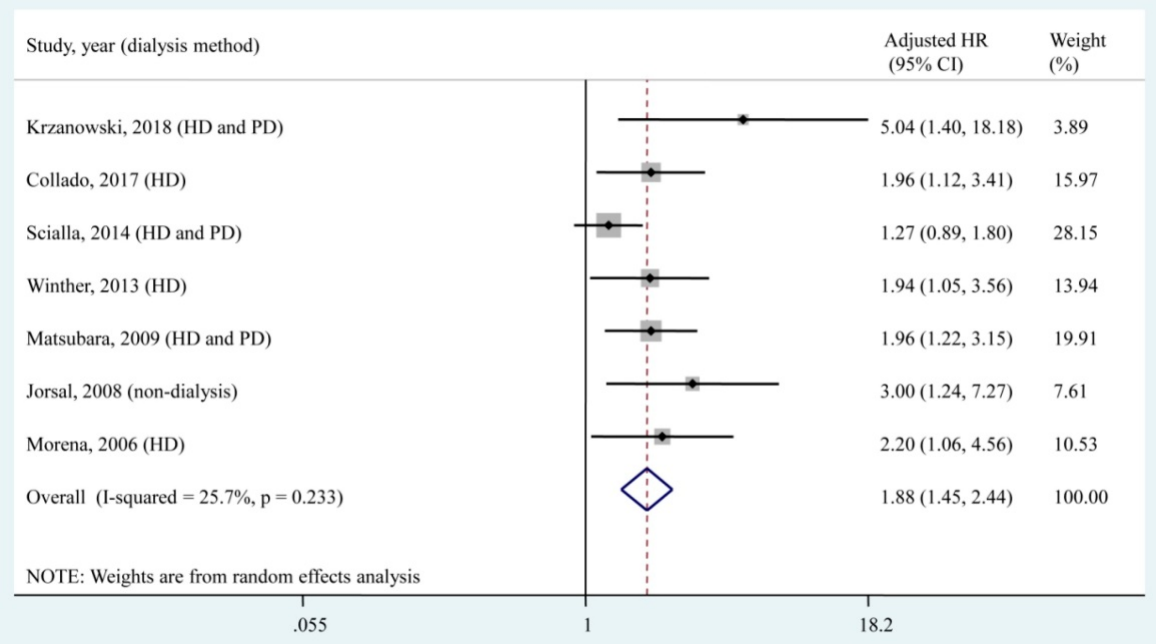
**Forest plot for the association of the circulating OPG level as a categorical variable with all-cause mortality.** HD, haemodialysis; PD, peritoneal dialysis; HR, hazard ratio; CI, confidence interval. The point estimates of adjusted HRs for each study are shown as solid boxes, and the size of each solid box indicates its weight in the analysis. Error bars are 95% CIs. The summary results are shown as solid prisms. 95% CIs are presented as the error bars or the width of the prisms. The summary adjusted HR was 1.88 (1.45, 2.44), with low heterogeneity (*I*² = 25.7%, *P* = 0.233).

Seven studies 24, 26-28, 30-32, which included a total of 1,934 patients, reported the adjusted HR of all-cause mortality for the highest OPG level group compared with the lower OPG level group. According to the qualitative meta-analysis, patients with the highest OPG levels had a significantly higher risk of all-cause mortality (adjusted HR, 1.88; 95% CI, 1.45 - 2.44) compared with patients with lower OPG levels, and low heterogeneity (*I*² = 25.7%, *P* = 0.233) was found among studies (**Fig. 2**).

Of the 7 included studies, 2 compared the high and low OPG levels according to the median value 24, 30, 3 compared the 3^rd^ tertile of the OPG level to the 1^st^ tertile 26-28, and 1 compared the 4^th^ tertile of the OPG level to the 1^st^ tertile 31. The 6 studies mentioned above set the lowest OPG level as the reference to assess the association between the highest OPG level and all-cause mortality. Only 1 study 32 set the middle OPG level (2^nd^ tertile) as the reference and found a significant increase in all-cause mortality associated with the highest OPG level (3^rd^ tertile, the adjusted HR, 2.20; 95% CI, 1.06 - 4.56) and a nonsignificant association with the lowest OPG level (1^st^ tertile, the adjusted HR, 1.52; 95% CI, 0.63 - 3.69).

**Fig 3:**
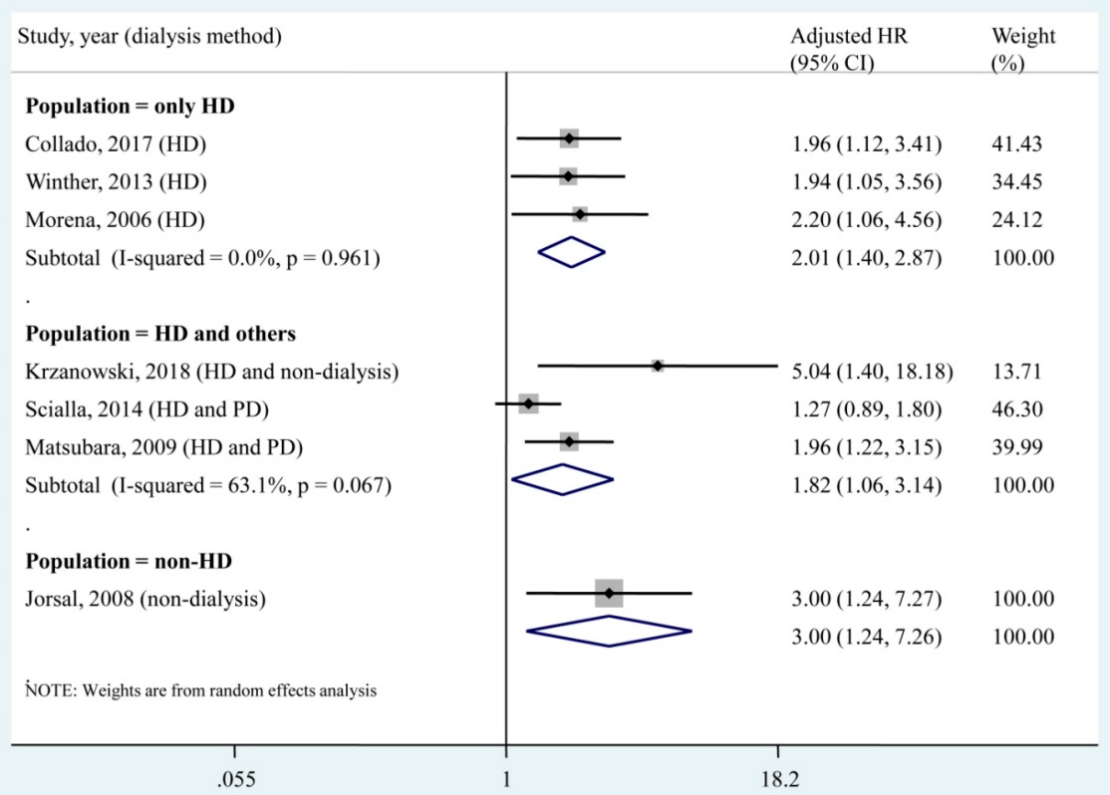
**Subgroup analysis according to dialysis methods for the association of circulating OPG level as a categorical variable with all-cause mortality.** HD, haemodialysis; PD, peritoneal dialysis; HR, hazard ratio; CI, confidence interval. The point estimates of adjusted HRs for each study are shown as solid boxes, and the size of each solid box indicates its weight in the analysis. Error bars are 95% CIs. The summary results are shown as solid prisms. 95% CIs are presented as the error bars or width of the prisms. The summary adjusted HR of the HD only population was 2.01 (1.40, 2.87), without heterogeneity (*I*² = 0, *P* = 0.961).

A subgroup analysis was conducted according to different dialysis methods (**Fig. 3**). The pooled HR of each subgroup demonstrated a significant association between the circulating OPG level and all-cause mortality. Specifically, for the population that underwent only haemodialysis (HD), no heterogeneity (*I*² = 0, *P* = 0.961) was found among studies.

### Association of a 1 pmol/L increase in the circulating OPG level with all-cause mortality

**Fig 4:**
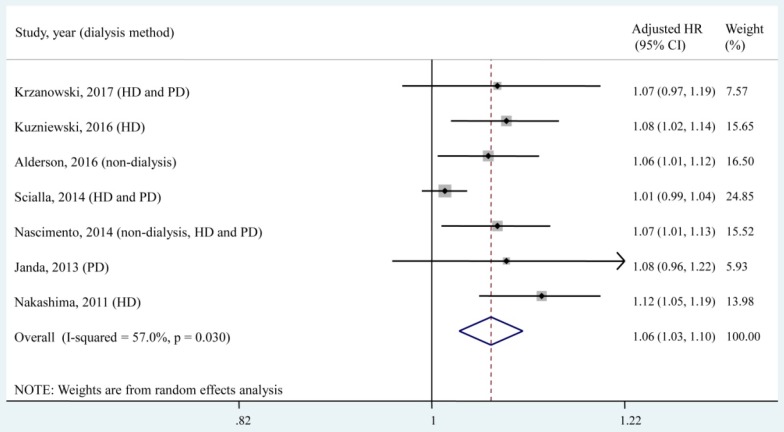
** Forest plot for the association of a 1 pmol/L increase in the circulating OPG level with all-cause mortality.** HD, haemodialysis; PD, peritoneal dialysis; HR, hazard ratio; CI, confidence interval. The point estimates of adjusted HRs for each study are shown as solid boxes, and the size of each solid box indicates its weight in the analysis. Error bars are 95% CIs. The summary results are shown as solid prisms. 95% CIs are presented as the error bars or width of the prisms. The summary adjusted HR was 1.06 (1.03, 1.10), with moderate heterogeneity (*I*² = 57.0%, *P* = 0.030).

Seven studies 8-10, 15, 25, 27, 29, which included a total of 1,563 patients, reported the adjusted HR of all-cause mortality for a 1 pmol/L increase in the circulating OPG level. According to the quantitative meta-analysis, each 1 pmol/L increase in the circulating OPG level was associated with a 6% increased risk of all-cause mortality (adjusted HR, 1.06; 95% CI, 1.03-1.10), and moderate heterogeneity (*I*² = 57.0%, *P* = 0.030) was found among studies (**Fig. 4**).

**Fig 5:**
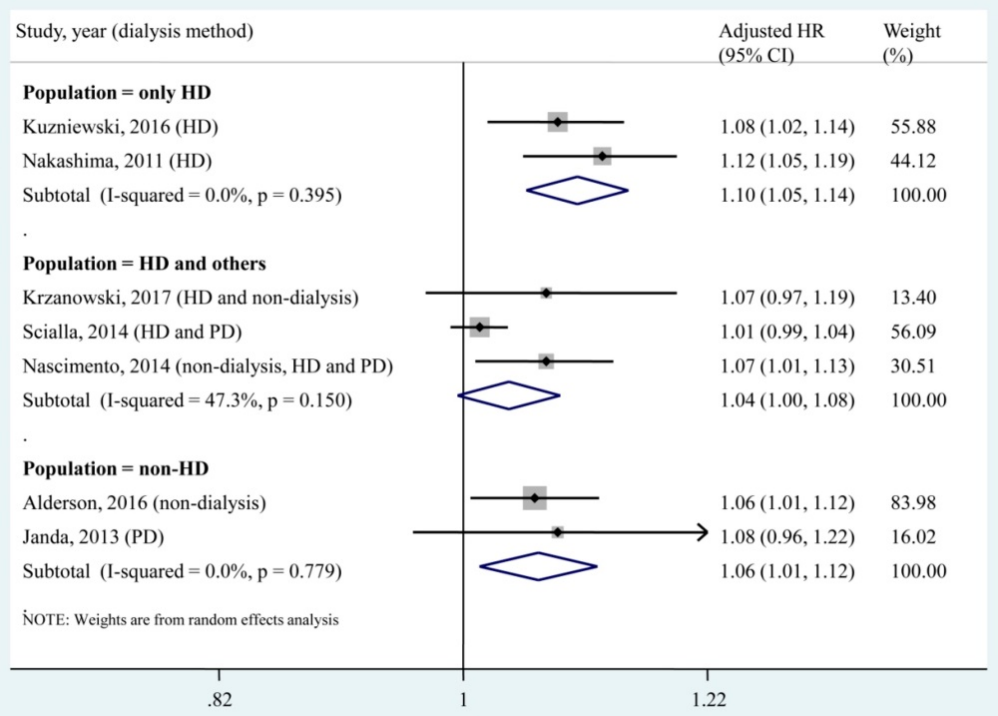
** Subgroup analysis according to dialysis methods for the association of a 1 pmol/L increase in the circulating OPG level with all-cause mortality.** HD, haemodialysis; PD, peritoneal dialysis; HR, hazard ratio; CI, confidence interval. The point estimates of adjusted HRs for each study are shown as solid boxes, and the size of each solid box indicates its weight in the analysis. Error bars are 95% CIs. The summary results are shown as solid prisms. 95% CIs are presented as the error bars or width of the prisms. The summary adjusted HR of the HD only population was 1.10 (1.05, 1.14), without heterogeneity (*I*² = 0, *P* = 0.395).

A subgroup analysis was conducted according to different dialysis methods (**Fig. 5**). We found that each 1 pmol/L increase in the circulating OPG level was significantly associated with increased risk of all-cause mortality in the population that underwent only HD (2 studies, adjusted HR, 1.10; 95% CI, 1.05-1.14). In addition, the pooled estimate of the subgroup including the HD population and others (adjusted HR, 1.04; 95% CI, 1.00-1.08) was obviously lower than that of the HD only subgroup.

### Sensitivity analysis

**Fig 6:**
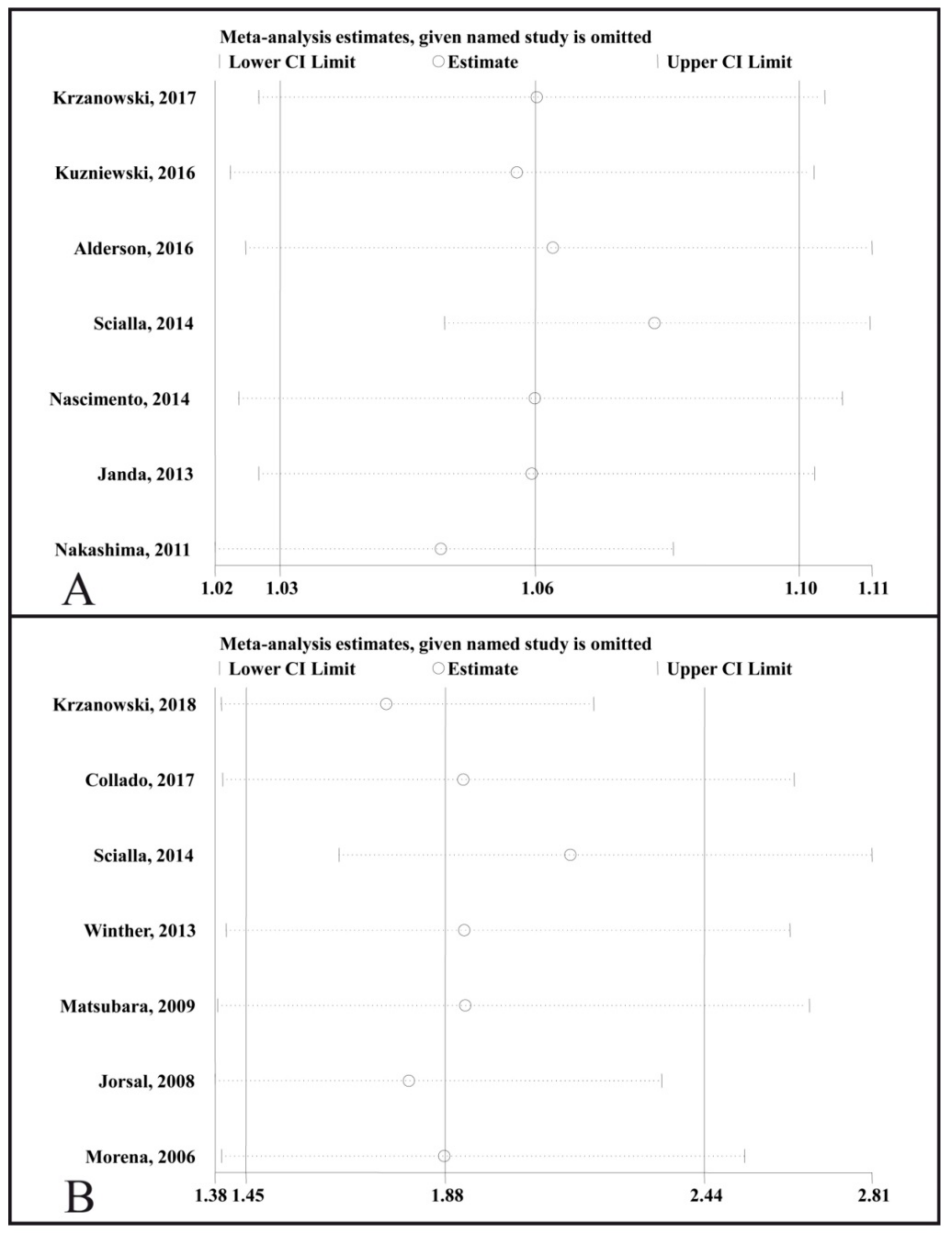
** Plot of sensitivity analysis by excluding one study at a time and the pooling hazard ratio for the remaining studies.** CI, confidence interval. (A), sensitivity analysis for the association of the circulating OPG level as a categorical variable with all-cause mortality. (B), sensitivity analysis for the association of a 1 pmol/L increase in the circulating OPG level with all-cause mortality.

A sensitivity analysis was performed by sequentially removing one study (**Fig. 6**). We found that the adjusted HR for all-cause mortality that compared the highest to the lowest circulating OPG levels was not significantly changed (**Fig. 6A**), and the adjusted HR for the change in all-cause mortality was not associated with a 1 pmol/L increase in the circulating OPG level (**Fig. 6B**).

## Discussion

The present meta-analysis examined the association of circulating OPG levels with all-cause mortality in CKD patients. The pooled results showed that a higher circulating OPG level was associated with a higher all-cause mortality risk in CKD patients (adjusted HR, 1.88; 95% CI, 1.45 - 2.44), with low heterogeneity (*I*² = 25.7%, *P* = 0.233). Each 1 pmol/L increase in the circulating OPG level was associated with a 6% increased risk of all-cause mortality (adjusted HR, 1.06; 95% CI, 1.03-1.10), with moderate heterogeneity (*I*² = 57.0%, *P* = 0.030). These pooled results suggested that OPG is an independent predictor of all-cause mortality in patients with CKD.

In 2008, Nybo and Rasmussen conducted a systematic review on the relationship between OPG levels and mortality 33. A quantitative summary was not performed due to methodological issues; nevertheless, the authors' findings supported the role of OPG as a predictor of cardiovascular disease and mortality. OPG is a soluble TNF superfamily receptor that has been implicated in changes in vessel matrix composition, the development of macroangiopathy, plaque destabilization and left ventricular hypertrophy 12, 34. OPG is secreted directly from the vascular wall, where it modulates apoptosis, inflammation, and calcium deposition 35. Additionally, its primary role may be in bone, where OPG is secreted by osteoblasts to inhibit the differentiation and maturation of neighbouring osteoclasts 35. A higher level of OPG likely indicates a compensatory increase in the local level of OPG in the vascular wall, which functions to counteract vascular calcium deposition. Alternatively, the higher OPG level may result from the transition of vascular smooth muscle cells to cells resembling osteoblasts. Together, these results imply a role of the active process of vascular calcification in the high risks of mortality and cardiovascular disease in CKD patients. Hence, the main implication of elevated OPG activity is the promotion and progression of atherosclerotic lesions, which might explain the significant association of OPG with mortality.

Dialysis was reported as a predictor of mortality in an end-stage renal disease (ESRD) population, but the survival benefit of one modality over the other has not yet been determined. One randomized controlled trial compared the mortality risk between the HD and PD modalities after 5 years of follow-up and found that the HD population suffered higher mortality than PD patients (HR, 3.8; 95% CI, 1.1 - 12.6) 36. Liem et al. also reported that the overall mortality was higher in patients treated with HD and in patients treated with PD 37. However, several observational studies reported different results in that patients who received the two dialysis modalities had similar mortality rates 38, 39 and that the PD modality led to a worse outcome than the HD modality 40, 41. A previous meta-analysis compared the two dialysis modalities in Korean patients and suggested a higher risk of death in elderly patients who received PD compared with those who received HD 42. These controversial results may be attributable to different baseline characteristics, which lead to interactions between the dialysis modality and mortality outcome. In the present meta-analysis, a subgroup analysis found that circulating OPG levels (as a categorical variable or a continuous variable) were significantly associated with all-cause mortality in the HD only population (**Fig. 3** and **Fig. 5**). This result supported OPG as an independent predictor of all-cause mortality in patients who underwent only HD. However, for the subgroup that included HD patients and others, no significant association was found between each 1 pmol/L increase in the circulating OPG level and all-cause mortality (adjusted HR, 1.04; 95% CI, 1.00-1.08). This result implied that each 1 pmol/L increase in the circulating OPG level may not be an independent predictor of all-cause mortality in non-HD patients. In the present meta-analysis, only 1 study 15 investigated the association between a 1 pmol/L increase in the circulating OPG level and all-cause mortality in a population that underwent only PD, and no significant result was found (adjust HR, 1.08; 95% CI, 0.96 - 1.22). Therefore, more studies should be performed to investigate the association between OPG and mortality in the PD only population.

The primary strength of the present meta-analysis was that the investigation of the relationship between the circulating OPG level and all-cause mortality considered OPG as not only a categorical variable but also a continuous variable. The pooled results showed that each 1 pmol/L increase in the circulating OPG level was associated with a 6% increased risk in all-cause mortality. In addition, a subgroup analysis according to the dialysis method suggested that an elevated circulating OPG level was an independent predictor of all-cause mortality in the HD only population. A previous meta-analysis showed that higher OPG levels were not significantly associated with higher all-cause mortality in HD patients, with a pooled HR of 1.80 (95% CI, 0.95 - 3.39) 43. However, this previous meta-analysis revealed high heterogeneity among studies (*I*² = 85.6%, *P* = 0.000) 43. Most importantly, this previous meta-analysis did not investigate the association of a 1 pmol/L increase in the level of circulating OPG with the risk of all-cause mortality 43.

This meta-analysis had several limitations. First, the studies included in this meta-analysis were essentially observational in nature; it was impossible to fully adjust for potential confounders, such as nutritional status and declining kidney function during follow-up. Second, when investigating the relationship between the circulating OPG level as a categorical variable and all-cause mortality, each study adjusted for different factors and had varied definitions and cut-off values for the OPG groups. Third, a relatively small number of studies was included in the meta-analysis. Thus, the funnel plots and Egger's test were not valid because the accuracy of these tests is low and may even be misleading when fewer than 10 studies are available for the quantitative summary 44. Fourth, in the quantitative analysis, the HD only subgroup had a higher HR than the other groups. The higher prevalence of classic risk factors and novel cardiovascular markers in HD patients compared with those in PD or non-dialysis patients may also serve as an important reason for this difference. More studies are needed to explore the impact of classic risk factors and novel cardiovascular markers in the relationship between the dialysis modality and mortality in CKD patients. Fifth, the present meta-analysis did not further explore the role of OPG in cardiovascular mortality or cardiovascular events in CKD patients. Cardiovascular death may be the leading cause of death in patients with CKD, and these patients have a 10-30 times higher cardiovascular mortality risk than the general population 45. A previous meta-analysis supported the predictive value of OPG for cardiovascular mortality in HD patients (adjusted HR, 2.53; 95% CI, 1.29 - 4.94) despite its heterogeneity 43. However, because our aim was to investigate the association of OPG and all-cause mortality in CKD patients, some studies that focused on cardiovascular mortality or cardiovascular events were not included in the present meta-analysis. Thus, it was not appropriate to analyse the possible role of OPG in cardiovascular mortality and cardiovascular events based on the included studies in the present meta-analysis. We plan to explore the association of OPG and cardiovascular mortality or cardiovascular events in our next study. Finally, selective reporting bias in the literature may have influenced the present findings.

Heterogeneity was low (*I*² = 25.7%, *P* = 0.233) for the qualitative meta-analysis but moderate (*I*² = 57.0%, *P* = 0.030) for the quantitative meta-analysis. This different result may be due to confounding variables (Table 1), which resulted in the adjusted HRs. The adjustment for potentially confounding variables varied largely across the included studies and included epidemiological characteristics, cardiovascular risk factors, biological laboratory variables and established biomarkers of mortality. To explore more evidence-based medical support for the relationship between OPG and mortality in CKD patients, harmonization of adjusted variables desirable for future research was performed.

## Conclusions

In conclusion, the present meta-analysis found that elevated circulating OPG levels independently predicted an increased risk of all-cause mortality in patients with CKD. Each 1 pmol/L increase in the level of circulating OPG was associated with a 6% increased risk of all-cause mortality. OPG potentially serves as an independent predictor of all-cause mortality in CKD patients, especially in the HD only population. The mechanism underlying this observation deserves further investigation, as does the predictive performance of OPG as a biomarker in the clinical setting.

## Figures and Tables

**Table 1:** Characteristics of 13 researches included in the meta-analysis

Author, year	Population	Dialysis method	Patients (n)	Male (n)	Age (years)	Osteoprotegerin (OPG)	Follow-up	Death (n)	Comparison	Adjusted HR (95% CI)	Quality score
Krzanowski, 2018^24^	Poland, stage 5	HD and non-dialysis	59	38	61 ± 16	median, 7.55 pmol/L	5 years	25	high vs. low (> median vs. ≤ median)	5.04 (1.40, 18.18)	6
Collado, 2017^26^	Spain, ESRD	HD	220	154	61.1 ± 6.1	8.78 (6.07-12.95) pmol/L	3.2 ± 1.91 years	74	high vs. low (Tertile 3 vs. Tertile 1)	1.96 (1.12, 3.41)	7
Krzanowski, 2017^25^	Poland, stage 5	HD and non-dialysis	78	46	NA	NA	5 years	27	per 1 pmol/L	1.07 (0.97, 1.19)	7
Kuzniewski, 2016^8^	Poland	HD	69	39	60 ± 12	13.33 (10.53-17.38) pmol/L	7 years	39	per 1 pmol/L	1.08 (1.02, 1.14)	6
Alderson, 2016^9^	CRISIS, stage 3-5	non-dialysis	463	286	63.8 ± 14.1	7.87 ± 3.28 pmol/L	46 (21 - 69) months	217	per 1 pmol/L	1.06 (1.01, 1.12)	6
Scialla, 2014^27^	CHOICE, ESRD	HD and PD	602	320	57.8 ± 14.9	10.9 (8.0-15.3) pmol/L	13.3 years	423	high vs. low (3^rd^ tertile vs. 1^st^ tertile)	1.27 (0.89, 1.80)	7
									per 5 pmol/L	1.07 (0.95, 1.20)	
Nascimento, 2014^10^	Brazil, stage 3-5	non-dialysis, HD and PD	145	88	median, 61	8.9 (1.89-33.2) pmol/L	3 years	40	per 1 pmol/L	1.07 (1.01, 1.13)	8
Winther, 2013^28^	Denmark, with established CVD	HD	206	133	67 ± 12	5.52 ± 3.18 ng/L	2 years	90	high vs. low (3^rd^ tertile vs. 1^st^ tertile)	1.94 (1.05, 3.56)	6
Janda, 2013^15^	Poland	PD	55	30	53 ± 13	NA	6 years	22	per 1 pmol/L	1.08 (0.96, 1.22)	7
Nakashima, 2011^29^	Japan	HD	151	85	62.1 ± 13.4	10.5 (7.3-15.1) pmol/L	6 years	40	per 1 pmol/L	1.12 (1.05, 1.19)	7
Matsubara, 2009^30^	Sweden, stage 5	HD and PD	265	165	53 ± 10	median, 2,035 pg/mL	5 years	84	high vs. low (> median vs. ≤ median)	1.96 (1.22, 3.15)	7
Jorsal, 2008^31^	Denmark, T1DM with nephropathy	non-dialysis	397	243	42.1 ± 10.6	3.0 (1.4-11.4) ng/mL	11.3 (0-12.9) years	126	high vs. low (4^th^ quartile vs. 1^st^ quartile)	3.00 (1.24, 7.27)	7
Morena, 2006^32^	France	HD	185	93	median, 66.7	median, 1894.2 pg/ml	2 years	50	high vs. low (3^rd^ tertile vs. 2^nd^ tertil)	2.20 (1.06, 4.56)	7
	
Author, year	Confounding variables										
Krzanowski, 2018^24^	Dialysis status, Framingham risk score, atherosclerotic plaques in CCA										
Collado, 2017^26^	Age, Charlson Comorbidity Index, smoking, albumin, IL-18, Troponin I										
Krzanowski, 2017^25^	Age, dialysis status, pentraxin 3, high‑sensitivity CRP										
Kuzniewski, 2016^8^	Dialysis duration, sex, diabetes mellitus, hypertension, smoking, LDL-cholesterol, CRP, albumin, PTH and Ca x Pi										
Alderson, 2016^9^	Age, sex, creatinine, prior cardiovascular event, heart failure at baseline, diabetes mellitus, current or former smoker, mean SBP, Pi, Ca, albumin, haemoglobin, PTH, FGF-23, fetuin-A										
Scialla, 2014^27^	Age, sex, race, index of coexistent disease, diabetes mellitus, cardiovascular disease, BMI, Pi, and corrected Ca, albumin, IL-6, CRP, FGF-23										
Nascimento, 2014^10^	Age, sex, high‑sensitivity CRP, albumin, diabetes mellitus										
Winther, 2013^28^	Age, sex, blood pressure, diabetes mellitus, Ca x Pi, albumin, fbrinogen, CRP, adiponectin, treatment with n-3 polyunsaturated fatty acids/placebo										
Janda, 2013^15^	Age, FGF-23, coronary arteries calcification score										
Nakashima, 2011^29^	Age, sex, dialysis duration, diabetes mellitus, baseline CVD										
Matsubara, 2009^30^	Age, sex, diabetes mellitus, CRP, CVD										
Jorsal, 2008^31^	Age, sex, smoking, blood pressure, Glycosylated Hemoglobin, GFR, serum cholesterol, UAER, antihypertensive treatment, cardiovascular events at baseline										
Morena, 2006^32^	Age, sex, dialysis duration, diabetes mellitus, hypertension, smoking										

OPG, osteoprotegerin; HR, hazard ratio; CI, confidence interval; HD, hemodialysis; PD, peritoneal dialysis; NA, data was not reported; CRISIS, The Chronic Renal Insufficiency Standards Implementation Study; CHOICE, Choices for Healthy Outcomes In Caring for ESRD study; ESRD, end-stage renal disease; CVD, cardiovascular disease; T1DM, type 1 diabetic mellitus; CCA, common carotid artery; CRP, C‑reactive protein; LDL, low density lipoprotein; PTH, parathyroid hormone; Ca, calcium; Pi, phosphate; SBP, systolic blood pressure; FGF-23, fibroblast growth factor-23; BMI, body mass index; CVD, cardiovascular disease; GFR, glomerular filtration rate; UAER, urinary albumin excretion rate
